# 

**Published:** 1893-10-28

**Authors:** 


**OUR NEW OFFICES,**
428, Strand, W.C., will henceforth be the Office of this Journal.

**Notices of Births, Deaths, and Marriages** are inserted in this column at a charge of 2s. 6d. for an announcement not exceeding four lines.

### NOTICE TO CORRESPONDENTS.

All MS., Letters, Books for Review, and other matters intended for the Editor should be addressed The Editor, The Lodge, Porchester Square, London, W.

The Editor cannot undertake to return rejected MS., even when accompanied by stamped directed envelope.

**Notice to Advertisers and Subscribers.**

All Advertisements, Orders for Copies of The Hospital, and Business Communications should be addressed to The Publisher, *not to the Editor*, The Hospital, 428, Strand, W.C.

The Cost of Subscription, post free, is as follows:—Per week, 2½d.; per quarter, 2s. 6d.; per half-year, 5s.; per annum, 8s. 6d. Foreign:—Per Annum, 10s. 8d.; payable, in all cases, in advance.

**"Burdett's Hospital Annual," 1893.**

Fourth year of publication. 700 pp. Boards, gilt lettering. A most complete guide to British, Colonial, Indian, and American Hospitals, Dispensaries, Nursing and Convalescent Institutions, and Asylums. Price 5s. Published by The Scientific Press (Limited), 428, Strand, W.C.

**NOTICE.—The Index for Vol. XIII. (ending March, 1893) is on sale at the Office of this Journal, price 2½d., post free.**

### Scraps and Gleanings.

The total amount now received on behalf of the Hospital Saturday Fund, for the present year, reaches £15,000.

Since the Cirencester Cottage Hospital was opened in 1875, 1,727 cases have been treated within its walls. It is feared that this year the receipts will scarcely cover the expenses.

It is asserted that cases have again occurred in Beckenham of infection transmitted by patients discharged from the Bromley and Beckenham Joint Hospital for Infectious Diseases.

An indignation meeting was held recently by the ratepayers of Aylestone (Leicester) to protest against the site selected in that locality for the erection of an infectious diseases hospital.

Mr. M'Calmont has contributed £250 towards the drainage works at Addenbrooke's Hospital, Cambridge; the committee has received £1,039 during the last quarter for the same object.

It is proposed to increase the accommodation for nurses at the Salisbury Infirmary and add a school for training nurses for house to house visitation. The cost has been estimated at £2,500.

The collections on Hospital Saturday at Colchester, which amounted to £150, were devoted to the Essex and Colchester Hospital. The day's proceedings terminated with a concert given by the Good Templars' Choir

The Friendly Societies of Leominster recently held their annual church parade in aid of the proposed cottage hospital for that town. Over 1,000 persons took part in the procession, and attended service at the Priory Church.

The number of patients treated at the Heston and Isleworth Infectious Diseases Hospital during the present year has been 77, a larger number than has been received since its opening in 1881. Only two deaths have occurred.

The Public Health and Housing Committee of the London County Council have reported in favour of acquiring ten acres of the Millbank Prison site for the purpose of erecting thereon house accommodation for about 3,700 persons.

The Committee of the Cottage Hospital, St. Helens, are again able to present a satisfactory balance-sheet. It has been found necessary to make an addition to the hospital at a probable cost of about £3,000, and the work has already been commenced.

A convalescent home, under the management of the Newcastle Cathedral Nurse and Loan Society, was recently opened at Hexham. The home is at present for women and children only, but it is hoped in time to enlarge it sufficiently to admit men also.

The twenty-fifth annual report of the committee of the Governesses' Convalescent Home, Southport, shows an expenditure of £591, and a balance in hand of £41. Considerable improvements have been made in the sanitation of the institution at a cost of £135.

The success of the collectors on Hospital Saturday at Cork, when £360 was realised, deserves unusually high praise, since the number of country people visiting Cork was fewer than on ordinary Saturdays owing to the races held at the beginning of the week.

Salisbury Infirmary recently celebrated its 127th anniversary. During the past year 1,011 in-patients and 3,972 out-patients have received treatment. A new accident ward has been added, but a debt remains on the building fund of £1,800, and the annual expenditure exceeds receipts by £500.

Religious intolerance has been permitted to interfere with the usefulness of St. Cross' Hospital, Rugby. Three of the nurses having become Roman Catholics, recently, a resolution was passed that no Romanist should be permitted to hold office in the hospital. The resolution has aroused great indignation.

The new cottage hospital for Johnstone, Renfrewshire, was recently opened by the donor, Mr. Bine Renshaw, M.P., who, besides providing for the cost of construction, has also given £3,000 towards the endowment fund, and promised £100 per annum, for five years, for current expenses. The hospital contains 20 beds.

The work of restoration at the Torbay Hospital and Infirmary has proved a greater source of expense than the committee had anticipated it would be, and it has now been seen that the work cannot be carried out within the original estimate; £1,850 is still required to enable the contractors to complete the extension.

A proposal to erect a workhouse infirmary at Skircoat, the residential suburb of Halifax, has met with strenuous opposition from the Halifax Town Council and residents of Skircoat, on the ground that the site is only capable of artificial drainage, and that such a building would depreciate the value of the land in the suburb.

An appeal has been issued in Guildford for more subscriptions for the Surrey Convalescent Home. While only 24 guineas are subscribed annually in the town, Guildford patients are using nominations to the value of about 100 guineas, exclusive of the Friendly Societies and firm's subscriptions, which benefit their own members.

### PUBLISHERS' ANNOUNCEMENTS.

Will be ready shortly. Crown 8vo, Cloth. Price 2s. 6d., post free.

NOTES ON NURSING IN EYE DISEASES.

Illustrated with Wood Engravings and Process Blocks.

By C. S. JEAFFRESON, M.D., F.R.C.S.E.,

Senior Surg. Northumberland, Durham and Newcastle Infirm. for Diseases of Eye; Hon. Cons. Ophth. Surg. Northumberland and Durham Soc. for the Blind, etc., etc.

Post free. 2/6 In handsome cloth boards, 150 pp., Illustrated. Post free. 2/6

Ministering Women:

The Story of the Royal National Pension Fund for Nurses.

By GEORGE W. POTTER, M.D.

"Is full of interest, and the sale of the book will be stimulated by the fact that the profits of the author are to be devoted to the 'Junius S. Morgan Benevolent Fund.' An excellent portrait of the Princess of Wales forms a suitable frontispiece."—*Liverpool Courier.*

"It should commend itself to all—women especially—who can appreciate the noble work undertaken by those true sisters of mercy and self-denial, our hospital nurses. The record is full of touching interest. . . . The book is well worth the half-crown charged. . . . Every nurse should possess herself of a copy."—*The Lady.*

London: The Scientific Press (Limited), 428, Strand, C.W.

QUESTIONS AND ANSWERS ON NURSING.

By Dr. J. W. MARTIN.

LEONARD'S MANUAL OF BANDAGING

With 130 Engravings, Price 3s. 6d.

THE HYGIENE OF BEAUTY.

By Dr. MONIN, of Paris.

Translated by Mrs. CARDWELL.

Price 3s. 6d.

Bailliere, Tindall, and Cox, King William Street, Strand.

*Catalogues Post Free.*

BOOKS ON NURSING *AND ALLIED SUBJECTS.*

The Scientific Press will be pleased to supply any books on the above subjects, post free, at the published price.

Special quotations given for the supply of Hospitals, Nursing Schools, and Libraries in quantities.

London: The Scientific Press (Limited), 428, Strand, W.C.

Now ready, crown 8vo., 5th Edition. Price 4s. net.

MANUAL OF OBSTETRIC AND GYNÆCOLOGICAL NURSING.

By FLEETWOOD CHURCHILL, M.D. Revised and Edited by THOMAS MORE-MADDEN, M.D., F.R.C.S.Ed. Obstetric Physician to Mater Misericordiæ Hospital; Master of the National Lying-in Hospital, Dublin.

"We can confidently recommend this revised work to all interested in the subject of which it treats."—*The Hospital.*

"Dr. More-Madden's Handbook . . . may be safely recommended as a reliable Text-book."—*Provincial Medical Journal.*

"Our opinion is that not only is it an excellent and complete work for the nurse's use, but many students and young practitioners would gain much that would be of use from its perusal."—*Medical Times and Hospital Gazette.*

Fannin and Co., 41, Grafton Street, Dublin. London: Bailliere, Tindall, and Cox.

Demy 8vo., Cloth, Illustrated, Price 2s. 6d.

Mental Nursing: A Text BOOK FOR ASYLUM ATTENDANTS AND ALL NURSES WISHING TO TAKE CHARGE OF MENTAL CASES. By WILLIAM HARDING, M.B., &c. The want of a complete book for the instruction of Asylum Attendants and Nurses has been long felt, and it is with confidence that the publishers recommend this work to the managers of all Institutions for the Insane.

London: The Scientific Press (Limited), 428, Strand, W.C.

### Books Received.

J. and A. Churchill.

"Materia Medica, Pharmacy, Pharmacology, and Therapeutics." By W. Hale White, M.D.

Cassell and Co.

"Tumours Innocent and Malignant." By J. Bland Sutton.

**Periodicals and Pamphlets.**—Letts' Improved Pocket Diary, Improved Octavo Diary, Waistcoat-pocket Diary, Improved Office Diary, Improved Diary, Nurses' Diary, and Improved Date Indicator, all for 1894.

**The Hospital Advertiser.**

**THE "NURSE."**

### A COMPLETE
### NURSE'S LIBRARY.

| | s. | d. |
|---|---|---|
| **THE THEORY AND PRACTICE OF NURSING:** | | |
| By Percy G. Lewis, M.D. | 3 | 6 |
| **SURGICAL WARD-WORK AND NURSING:** | | |
| By Alexander Miles, M.D. | 3 | 6 |
| **OPHTHALMIC NURSING:** | | |
| By Sydney Stephenson, M.B. | 3 | 6 |
| **MENTAL NURSING:** | | |
| By William Harding, M.B. | 2 | 6 |
| **THE NURSE'S DICTIONARY:** | | |
| By Honnor Morten | 2 | 0 |
| **THE ART OF FEEDING THE INVALID:** | | |
| By A Medical Practitioner | 3 | 6 |
| **A PRACTICAL HANDBOOK OF MIDWIFERY:** | | |
| By Francis W. N. Haultain, M.D. | 5 | 0 |
| **THE ART OF MASSAGE:** | | |
| By A. Creighton Hale | 6 | 0 |
| **HOSPITAL SISTERS AND THEIR DUTIES.** | | |
| By Eva C. E. Lückes | 2 | 6 |
| **HOW TO BECOME A NURSE:** | | |
| By Honnor Morten | 2 | 6 |
| **THE NURSE'S CASE-BOOK:** | | |
| For Hospital and Private Uses | 0 | 6 |

**The Complete Set, Carriage Paid, £1 10s.**

**CATALOGUES SENT POST FREE TO APPLICANTS.**

London: The Scientific Press, Limited, 428, Strand, W.C.

### PUBLISHERS' ANNOUNCEMENTS.

**"MR. BURDETT'S MONUMENTAL WORK."—THE TIMES.**

In FOUR VOLUMES: Crown quarto, handsome cloth gilt, bevelled edges, PROFUSELY ILLUSTRATED with PLANS and DESIGNS. Together with a portfolio containing SOME HUNDREDS OF PLANS. Price £8 8s. complete:—

### HOSPITALS AND ASYLUMS OF THE WORLD.

Their Origin, History, Construction, Administration, Management, and Legislation; with Plans of the Chief Hospitals, Asylums, Convalescent Institutions, Special and Infectious Hospitals, Nurses, Homes, and Medical School Buildings, in addition to those of all the Hospitals of London in the Jubilee Year of Queen Victoria's Reign, Accurately drawn to a Uniform Scale.

**By HENRY C. BURDETT.**

Formerly Secretary and General Superintendent of the Queen's Hospital, Birmingham, and Registrar of the Medical School; The "Dreadnought" Seamen's Hospital, Greenwich; Founder of the "Home Hospitals for Paying Patients," The Hospitals Association, and the Royal National Pension Fund for Nurses; Author of "Pay Hospitals of the World," "Hospitals and the State," "Cottage Hospitals: General, Fever, and Convalescent with Fifty Beds and Under," "The Relative Mortality of Large and Small Hospitals," "Helps to Health," "Burdett's Hospital Annual and Year Book of Philanthropy"; and Editor of The Hospital.

**VOLS. I. and II.—ASYLUMS—HISTORY and ADMINISTRATION, CONSTRUCTION, PLANS, and BIBLIOGRAPHY. Price £4 10s.**

**VOLS. III. and IV.—HOSPITALS—HISTORY and ADMINISTRATION, CONSTRUCTION, PLANS and BIBLIOGRAPHY. With Portfolio containing some Hundreds of Plans. Price £6.**

**THE ENTIRE WORK COMPLETE, Price £8 8s.**

**SPECIAL NOTE TO BOOK BUYERS, LIBRARIANS, AND THE TRADE.**

ONLY A LIMITED NUMBER OF COPIES OF THIS WORK WERE PRINTED FOR SALE, MOST OF WHICH ARE ALREADY DISPOSED OF.

"All honour to Mr. Burdett that he has erected a monument more enduring than brass."—*The British Medical Journal.*

"These magnificent volumes . . . . the outcome of a vast amount of laborous investigation and of many journeys in Europe, America, and the British Colonies."—*The National Observer.*

"Forming in itself a very valuable work of reference to the architect and all managers of hospitals."—*The Building News.*

London: THE SCIENTIFIC PRESS (Limited), 428, Strand, W.C.

### Medical Vacancies and Appointments.

### APPOINTMENTS.

J. Atlee, M.B., B.C.Cantab., appointed Ophthalmic House-Surgeon to St. Bartholomew's Hospital. W. Banks, M.B.Lond., M.R.C.S.Eng., reappointed Honorary Surgeon to the Falmouth Public Dispensary. D. Berry, appointed *pro tem.* Medical Officer of Health to the Burnham Local Board. R. A. Birdwood, M.D., M.R.C.S.Eng., appointed Medical Superintendent to the Metropolitan Asylums District. R. Boyle, L.R.C.P., L.R.C.S.Edin., L.F.P.S.Glasg., appointed Medical Inspector to the Universal White Lead Works, Millwall, London. E. C. Bridges, appointed Senior House-Physician to St. Bartholomew's Hospital. H. L. Brooksbank, M.B.Cantab., appointed Junior House-Physician to St. Bartholomew's Hospital. A. Buck, appointed Senior House-Surgeon to St. Bartholomew's Hospital. F. C. Bullmore, M.R.C.S.Eng., L.S.A., reappointed Honorary Surgeon to the Falmouth Public Dispensary. W. K. Bullmore, M.D.St. And., M.R.C.S.Eng., reappointed Acting Surgeon to the Falmouth Public Dispensary. C. Buttar, M.B.Cantab., appointed Senior House-Surgeon to St. Bartholomew's Hospital. A. Croll, M.B., C.M., appointed Resident Medical Officer to the Dundee Parochial Hospital. E. J. Cross, M.R.C.S.Eng., L.R.C.P.Lond., D.P.H. Camb., appointed Medical Officer for the Southwark District of the Fareham Union. A. R. Cushny, M.D.Aberd., appointed Professor of Materia Medica and Therapeutics in the University of Michigan, U.S.A. D. S. Davies, M.D., L.R.C.P.Lond., M.R.C.S.Eng., reappointed Medical Officer of Health to the Bristol Port Sanitary Authority. C. S. De Segundo, M.B.Lond., appointed Junior House-Physician to St. Bartholomew's Hospital. H. M. Evans, M.B.Lond., M.R.C.S., L.R.C.P., appointed Assistant Registrar at the Central London Throat, Nose, and Ear Hospital, Gray's Inn Road, P. Furnivall, appointed Junior House-Surgeon to St. Bartholomew's Hospital. A. B. Harris, M.D.St. And., L.R.C.P.Edin., M.R.C.S.Eng., reappointed Honorary Surgeon to the Falmouth Public Dispensary. J. C. Heaven, L.R.C.P.Lond., M.R.C.S.Eng., reappointed Assistant Medical Officer of Health to the Bristol Port Sanitary Authority. T. A. Helme, M.D., F.R.S.E., appointed Honorary Assistant Surgeon to the Clinical Hospital for Women and Children, Manchester, H. L. Hepburn, M.B.Lond., appointed Senior House-Surgeon to St. Bartholomew's Hospital. N. H. Hobart, M.B.Cantab., appointed Junior Resident Anæsthetist to St. Bartholomew's Hospital. C. Hodgson, M.R.C.S., L.R.C.P.Lond., appointed Assistant House-Surgeon to the County Hospital, York. W. J. Horne, appointed Junior House-Physician to St. Bartholomew's Hospital, P. Horton-Smith, M.B.Cantab., appointed Junior House-Physician to St. Bartholomew's Hospital. L. Jones, F.R.C.S., appointed Junior House-Surgeon to St. Bartholomew's Hospital. W. B. Jones, M.B.Lond., appointed Senior House-Surgeon to St. Bartholomew's Hospital. J. McGregor, M.B., C.M.Glasg., appointed House-Physician to the East London Children's Hospital, Shadwell, London, E. W. H. Maidlow, F.R.C.S., appointed Junior House Surgeon to St. Bartholomew's Hospital. J. Miller, appointed Senior House-Physician to St. Bartholomew's Hospital. G. B. Millett, L.R.C.P.Edin., M.R.C.S.Eng., reappointed Medical Officer of Health to the Penzance Town Council. E. H. Moore, L.R.C.S.Edin., L.S.A., reappointed Honorary Surgeon to the Falmouth Public Dispensary. E. Morse, L.R.C.P., L.R.C.S.Edin., reappointed Medical Officer of Health for Great Torrington. J. F. H. Owen, L.R.C.P.Edin., M.R.C.S.Eng., reappointed Honorary Surgeon to the Falmouth Public Dispensary. H. J. Paterson, M.B.Cantab., appointed Senior House-Surgeon to St. Bartholomew's Hospital. A. R. Phillips, L.D.S.R.C.S.Eng., reappointed Honorary Dental Surgeon to the Falmouth Public Dispensary. H. W. Pomfret, M.D.Vict., L.R.C.P.Lond., F.R.C.S.Eng., reappointed Medical Officer of Health to the Hollingworth Local Board. R. C. M. Pooley, L.R.C.P., L.R.C.S.I., reappointed Honorary Surgeon to the Falmouth Public Dispensary. G. P. Shuter, M.B.Cantab, appointed Senior Resident Anæsthetist to St. Bartholomew's Hospital. J. Smith, M.A., M.D.Edin., M.R.C.S.Eng., appointed Medical Officer to the Post Office, Kirkcaldy, N.B. Mr. R. Smith appointed Junior House Surgeon to St. Bartholomew's Hospital. E. H. E. Stack, M.B., B.C.Cantab., appointed Midwifery Assistant to St. Bartholomew's Hospital. C. Stein, M.D.Edin., appointed Medical Officer of the Workhouse of the Shipston-on-Stour Union. H. D. Stubbs, M.B.Cantab., appointed Extern Midwifery Assistant to St. Bartholomew's Hospital. J. Symington, M.D., F.R.C.S. Edin., appointed Professor of Anatomy in the Queen's College, Belfast. T. B. Thorne, M.B., B.S.Durh., appointed Senior House-Physician to St. Bartholomew's Hospital. W. H. Thompson, M.D.R.U.I., M.Ch., F.R.C.S.Eng., appointed Professor of Physiology in the Queen's College, Belfast. H. Troutbeck, M.B., B.C.Cantab., appointed Senior House Physician to St. Bartholomew's Hospital. W. A. Wetwan, M.R.C.S.Eng., L.S.A., reappointed Medical Officer of Health for the Bridlington Urban Sanitary District. C. Wiggens appointed Medical Officer of Heath for the Bexhill Urban Sanitary District. J. C. O. Will, M.D.Aberd., appointed Extra-professorial Examiner in Surgery for Degrees in Medicine at the University of Aberdeen. Mr. N. O. Wilson appointed Junior House-Surgeon to St. Bartholomew's Hospital. T. Wilson, L.R.C.P.Edin., M.R.C.S.Eng., reappointed Medical Officer of Health for Wallsend. B. C. A. Windle, M.D., D.Sc.Dub., appointed Extra-professorial Examiner in Anatomy for Degrees in Medicine at the University of Aberdeen. J. C. Wood, L.R.C.S. & P., D.P.H.Edin., reappointed Medical Officer of Health and Public Analyist for the County Borough of Sunderland.

### VACANCIES.

### Resident Medical Officers.

Belgrave Hospital for Children, 77 and 79, Gloucester Street, S.W. Physician for Outpatients. Applications to Honorary Secretary by November 8th.

City of Sheffield. Resident Medical Officer for the City Hospital for Infectious Diseases; doubly qualified. Salary £200 per annum, with board, lodging, and attendance. Applications endorsed "Medical Superintendent" to J. W. Pye-Smith, Town Clerk, Town Clerk's Office, Sheffield, by October 28th.

Dundee Royal Lunatic Asylum. Resident Clinical Assistant. Applications to Dr. Rorie, Westgreen House, Dundee.

### Non-Resident Medical Officer.

Charing Cross Hospital. Surgeon Dentist, must be M.R.C.S.Eng. Applications to Arthur E. Reade, Secretary, by October 31st.

### SIR EDWARD FRY ON COMPETITIVE EXAMINATIONS.

At the distribution of the Bristol Medical School, Sir Edward Fry said his first duty would be to congratulate those who had been successful in the competitions for which prizes and certificates had been awarded that day. He trusted the success of that day might be an augury to every one of success in practice and in life in the future. (Applause.) To those who had really striven to do their best and had failed in the competition to which he had referred, one had only to offer words of consolation and to advise them to remember that success in competition was by no means a test of success in life; that the capacity to win in a competitive examination was by no means the same thing as a win in the greater competition of life, and that there were a great many men who were not distinguished in the earlier portion of their career, but who had, nevertheless, developed great powers later in life. Although success was a subject for congratulation, failure was not a ground for disheartening anyone who had failed. For his own part he took part in the proceedings of that distribution with some amount of hesitation. He was alive to the advantages resulting from competitive examinations in many ways. He knew that it was no small thing that a man should have the capacity to acquire, perhaps, in a short space of time, a considerable amount of knowledge, and that he should be able to reproduce it with exactitude and precision, and he was alive to the fact that with increasing competitive examinations there must be a corresponding increase in the education of the country. (Applause.) But there were also some evils attendant upon the system of competitive examinations. In the first place it was to be observed that by some mysterious law of their constitution the purpose with which knowledge was acquired affected the retention of that knowledge. A man who desired to learn for the sake of knowing was much more likely to retain what he learnt than a man who learnt for the purpose of using that knowledge at a definite period—at an examination. It seemed as if the object in which a man set to work and the will mingled with the intellectual operations of the mind in acquiring knowledge. Knowledge acquired to be used at a definite time was apt to pass away when that occasion had gone by, but knowledge acquired with the desire really to know was, as it were, fixed in the mind. Therefore it was extremely important that students should study, not for the sake of producing it at some examination, but for the sake of knowing for its own worth. Another evil which, in his opinion, was apt to follow competitive examinations was the collapse of mind that was apt to follow the moment the student had got through his examination. A man who was a zealous student up to the last moment made no further advance in knowledge, and the mind seemed wearied with the effort it had made, and fell back upon the lower condition of intellectual aptitude. Further than that, if they substituted for the desire to know the desire to pass an examination, they were putting in the place of a permanent motive, which was an unlimited motive, a temporary motive, which was a very limited motive. These were attendant evils against which they should strive.

### PUBLISHERS' ANNOUNCEMENTS.

LONDON: THE SCIENTIFIC PRESS, LIMITED.

